# TNFAIP8: Inflammation, Immunity and Human Diseases

**Published:** 2019

**Authors:** Suryakant Niture, John Moore, Deepak Kumar

**Affiliations:** 1 Julius L. Chambers Biomedical Biotechnology Research Institute, North Carolina Central University Durham, NC 27707, USA

**Keywords:** TNFAIP8, Inflammation, Immunity

## Abstract

Tumor necrosis factor (TNF)-alpha-induced protein 8 (TNFAIP8 /TIPE) family proteins are known to be involved in maintaining immune homeostasis. The TIPE family contains four members: tumor necrosis factor-α-induced protein 8 (TNFAIP8), TNFAIP8 like 1 (TIPE1), TNFAIP8 like 2 (TIPE2), and TNFAIP8 like 3 (TIPE3). Here we review the latest roles and associations of a founding member of TIPE family protein - TNFAIP8 in cellular function/signaling, inflammation, and immunity related human diseases.

## Introduction

Inflammation can be caused by various environmental factors, including microbial infection and toxic chemical exposure. In response to inflammation, immune cells like macrophages, B and T lymphocytes, fibroblasts, endothelial cells, and various stromal cells secrete soluble polypeptide cytokine Tumor Necrosis Factor Alpha (TNFα) [1]. Dysregulation of the production of cytokine TNFα has been associated with the development of inflammatory bowel disease [2,3] psoriasis [3], major depression [4], Alzheimer’s disease [5], cancer [6,7] and other human diseases. Because of the importance of TNFα in such wide-ranging processes, the TNFα signaling pathway is being extensively studied and provides a rich source of potential targets to modulate disease outcomes.

Importantly, TNFα regulates the expression of a novel protein family called the tumor necrosis factor-α-induced protein 8 (TNFAIP8/TIPE) family. The TIPE family includes TNFAIP8, TNFAIP8-like 1 (TIPEl), TNFAIP8-like 2 (TIPE2), and TNFAIP8-like 3 (TIPE3) proteins. TNFα induction of TIPE proteins is initiated when TNFα binds with the TNFI/II receptor at the cell surface, leading to activation and nuclear localization of NF-kβ and binding to the target genes [8,9].

The complex biological roles of the TIPE family members in immunology are still in the early stages of discovery. Though specific roles for TIPE1 and TIPE2 have been delineated in the regulation of immunity, TNFAIP8 and TIPE3 have mainly been studied in models of tumorigenesis. Recently, we and others have analyzed the detailed molecular roles of TNFAI8 in human cancer [10,11], providing a starting point for investigation the role of TNFAIP8 in other conditions. In the current review, we focus on the role of TNFAIP8 in cell signaling inflammation, infections, immune regulation, and related diseases.

## The TIPE/TNFAIP8 Family

TIPE proteins are structurally similar and show ~75% of amino acid sequence similarity to each other, sharing a highly conserved C-terminal region and a more variable N-terminus [10]. All TIPE family members share a highly conserved hydrophobic TIPE2 homology (TH) domain and a death effector domain (DED) in their structure [12–17]. TIPE family proteins also feature a large central hydrophobic cavity surrounded by seven cylindrical a helices [10,16,17] and a deep central hydrophobic cavity which binds several potential phospholipid or lipid messengers [16–18].

There has been much recent interest in the roles that TIPE proteins play in TNFα regulated cellular processes, especially since they have been strongly associated with tumorigenesis and immunity. Interestingly, despite the existence of a significant sequence homology among the four members of this family, they are involved in different biological activities and exhibit remarkable variability of expression [10,11,19]. For example, several reports suggest that TNFAIP8 and TIPE3 proteins promote cell survival and drug resistance through blocking apoptosis [8,12], whereas TIPE1 and TIPE2 have been implicated in induction of apoptosis [20,21]. Thus, the TIPE proteins seem to play highly diverse and distinct roles in various physiological processes, depending on tissue and cellular context.

We have recently reviewed the current state of knowledge of the TIPE family of proteins [10], which highlighted the key molecular features and current biological roles attributed to the proteins. The different roles of the TIPE proteins at this early stage most likely reflect the models that have been emphasized for their individual study. For example, initial studies on TNFAIP8 have focused on its role in tumorigenesis. In these studies, TNFAIP8 has been shown to be oncogenic and enhance cell proliferation, tumor growth, and metastasis through induction of autophagy and by inhibition of apoptosis [10,22]. In immune system models, TIPE1 and TIPE2 negatively regulate innate and cellular immunity and have been linked to play an important role in inflammatory diseases [14,23–25], and TIPE3 has been shown to be involved in binding and transporting phosphoinositide second messengers [16]. As more functional studies are carried out in diverse model systems, a better perspective of the unique role each plays in immunity and related diseases will become more evident.

## Tumor Necrosis Factor Alpha Induced Protein 8 (TNFAIP8)

Human TNFAIP8 was first identified through comparison of primary and metastatic head and neck squamous cell carcinoma [26], and later TNFAIP8 was identified from endothelial cells as a TNFα inducible gene [27]. Genome mapping indicated that human *TNFAIP8* gene is located at the p23 region on chromosome 5 [10]. Expression of TNFAIP8 transcripts is found in most human cells and tissues including bone marrow, immune cells, adipose tissue, GI tract, lung, pancreas, placenta, salivary and thyroid glands, kidney, liver, ovary, and prostate tissues and TNFAIP8 proteins expression is induced in response to cellular inflammation mediated by TNFα [28,29]. Regulation of this gene may also be controlled by several other transcriptional factors, such as nuclear factor-Kb (NF-kβ), androgen receptor (AR), p53 and orphan nuclear receptor chicken ovalbumin upstream promoter transcription factor I (COUP-TFI) [8,22,30–32]. Human *TNFAIP8* gene encodes eight transcript variants/isoforms, whereas only five protein variants reported so far [10] and TNFAIP8 variant two predominantly expressed in prostate, breast, liver, lung cancer cells, and acute monocytic leukemia derived THP1 cells compared to other variants [10,33].

## TNFAIP8 Cell Signaling and Molecular Functions

Studies of TNFAIP8 in tumorigenesis has provided information on cellular pathways where TNFAIP8 plays a role [34]. Expression of TNFAIP8 in breast cancer cells MDA-MB-435 increases cell growth/metastasis and reduces cell apoptosis by increasing expression of VEGFR-2, MMP1, and MMP9 [12,13]. TNFAIP8 regulates Hippo signaling in lung and liver cancer cells by interaction with LATS1, and expression of TNFAIP8 induces cell proliferation, migration, invasion, and xenograft tumor growth [35,36]. In lung cancer cells, TNFAIP8 variant 2 (v2) regulates p53 signaling by controlling the expression and function of p53 protein [33]. Knockdown of TNFAIP8 v2 induces p53-independent inhibition of DNA synthesis, widespread p53 binding, the initiation of p53-dependent cell-cycle arrest, and sensitization of cells to DNA damaging reagents [33]. Mutant p53 (p53-K120) binds with the *TNFAIP8* locus at a cryptic p53 response element that is not occupied or bound by wild-type p53 and thus increases TNFAIP8 expression, which leads to enhanced lung cancer cell survival/proliferation [32]. In non–small cell lung carcinomas (NSCLC), TNFAIP8 knockdown inhibits EGF and IGF-1 stimulated migration in NSCLC cells by decreasing EGFR levels and by increasing sorting nexin 1 (SNX1), a key regulator of the EGFR trafficking protein [37]. TNFAIP8 expression is strongly associated with MMP9 and Ki-67 expression in endometrial tumor cells [38] and depletion of TNFAIP8 in esophageal squamous cell carcinoma cells induced cisplatin mediated apoptosis [39]. Similarly, depletion of TNFAIP8 in HeLa cervical cancer cells activates caspase-8/−3 and p38 phosphorylation and promotes cisplatin-induced cellular apoptosis and death [40]. In prostate cancer cell lines, TNFAIP8 interacts with ATG3 protein and induces autophagy and drug resistance and survival [22]. TNFAIP8 mediates oncogenic transformation in Balb-D2S cells through interaction with Gαi [41].

TNFAIP8 has also been investigated for its effects on gene expression. Depletion of TNFAIP8 in prostate cancer cells increases anti-proliferation and apoptosis-related genes such as *IL24, FAT3, LPHN2,* and *EPHA3,* fatty-acid oxidation gene, ACDL, and decreases the expression of several oncogenes including *NFAT5, MALAT1, MET, FOXA1, KRAS, S100P* and *OSTF1* [8]. These studies support the notion that TNFAIP8 plays distinct roles depending on tissue and cellular context.

## The Role of TNFAIP8 in Inflammation, Infection, Immunity and Related Human Diseases

The study of TNFAIP8 in cancer cells has established some basic features of TNFAIP8 biology, but how these processes differ in other cells, such as immune cells has not been fully established. Several recent studies suggest a potential role of murine TNFAIP8 in antibacterial immunity and in the inflammatory response. A microarray analysis showed that human macrophages stimulated with the TLR4 ligand LPS induced the expression of TNFAIP8 vi and v2 and displayed different kinetics and knockdown of TNFAIP8 v2 in A549 cells, in response to LPS induced expression of pro-inflammatory cytokines (IL-6, IL-8 and IL-1b, and TNFα). This suggests that TNFAIP8 v2 regulates anti-inflammatory pathways in resting and TLR ligand-stimulated cells [42]. This study emphasizes the need to dissect the roles of the different TNFAIP8 variants in immune responses.

The biological role of TNFAIP8 has also been investigated in bacterial *Listeria monocytogenes* infection [43]. TNFAIP8 regulates L. monocytogenes infection by inhibiting Ras-related C3 botulinum toxin substrate 1 (RAC1), which is involved in bacterial *L. monocytogenes* infections, by controlling pathogen invasion and host-cell apoptosis. The study showed that TNFAIP8-knockout mice are resistant to lethal *L. monocytogenes* infection and have a decreased bacterial load in the liver and spleen [43]. Infection of human mammary tumor cells or canine- derived adenofibrosarcoma cells with canine distemper virus decreased cell proliferation, and induced apoptosis/necrosis and mitochondrial membrane depolarization by increasing expression of *TNFAIP8* and *CDVM* gene expression, suggesting that TNFAIP8 can induce cell death in human mammary tumor cells infected with canine distemper virus [44].

TNFAIP8 expression is higher in lymphoid tissues and in the placenta, suggesting that TNFAIP8 may play other roles in modulating inflammation and immunity. Recently the effect of TNFAIP8 on cell mediated immunity of a cluster of differentiation (CD)4^+^ T lymphocytes in a cecal ligation and puncture (CLP) murine model was investigated [45], and the study demonstrated that expression of TNFAIP8 promotes CD4^+^ T lymphocyte proliferative activity *in vitro.* The expression of TNFAIP8 also affected splenic CD4^+^ T lymphocyte polarization following CLP induced sepsis *in vivo*, suggesting that TNFAIP8 modulates the pathogenesis of immune dysfunction in splenic T lymphocytes in mice [45]. Glucocorticoids are known to induce cell apoptosis and affect many human physiological systems, including nervous, skeletal, muscular, endocrine, circulatory, and the immune system [46], and a recent study demonstrated that TNFAIP8 facilitated glucocorticoid mediated cell apoptosis in mouse thymocytes [47].

Recently, the role of TNFAIP8 in acute Graft Versus-Host Disease was investigated in a murine model, and the study revealed that TNFAIP8 deficiency in allogeneic C57BL/6 recipient mice have a lower survival rate compared with allogeneic wild type recipients. TNFAIP8 deficiency increased splenic CD4^+^ cells levels, serum cytokines (IL-17A, TNF, and IL-6) levels, and active caspase-3 expression in the small intestine, whereas, cell survival factor Ki-67 expression was significantly decreased in epithelial cells of small intestine suggesting that TNFAIP8 might be involved in increased GI tract pathology risk [48]. Moreover, the expression of TNFAIP8 and TIPE2 is associated with diabetic nephropathy in glomeruli from streptozotocin (STZ)-induced diabetic rats, and renal biopsies of diabetic patients *in vivo* [49]. The study further revealed that TNFAIP8, and not TIPE2 expression, is upregulated in response to high glucose in mesangial cells which leads to increased cell proliferation and up-regulation of NADPH oxidase-mediated signaling pathway, suggesting that TNFAIP8 modulates diabetic nephropathy [49]. Indeed, although TNFAIP8 was initially described as a key regulator of cancer singling and tumorigenesis, recent reports suggest that TNFAIP8 also modulates inflammation, bacterial and viral infections, immune function and homeostasis in several disease conditions.

## Conclusions and Perspectives

TNFAIP8 has been extensively characterized in oncogenesis and has been shown to regulate cancer cell signaling resulting in increased drug resistance, cell proliferation, cell survival, cell metastasis, and autophagy. But new data is emerging that is expanding the role that TNFAIP8 plays in inflammation, bacterial and viral infections, immune function, and homeostasis in several disease conditions. However, the exact function of TNFAIP8 in regulation of immune response in chronic inflammatory diseases is still unknown. The biological role of TNFAIP8 in modulation of inflammation, alteration of immune response and regulation of cell survival or death appears to depend on the cellular and disease context. Several areas are ripe for further investigation. The role of the multiple TNFAIP8 variants in immune function and the biological significance of TNFAIP8 complexed with phospholipid or fatty acid in immune response are two examples. This area of investigation is likely to uncover the novel roles for TNFAIP8 in immune diseases, and potential new strategies to modulate immune disease.
